# S192. AKT-MTOR SIGNALING PATHWAY IS DOWNREGULATED IN SCHIZOPHRENIA

**DOI:** 10.1093/schbul/sby018.979

**Published:** 2018-04-01

**Authors:** Radhika Chadha, James Meador-Woodruff

**Affiliations:** The University of Alabama at Birmingham

## Abstract

**Background:**

Cognitive deficits are observed in many schizophrenia (SZ) patients. The AKT-mTOR pathway is an important signaling cascade associated with long term plasticity and thus may contribute to cognitive dysfunction. This pathway is tightly regulated by differential phosphorylation of key proteins. AKT is a serine-threonine kinase which regulates critical cellular functions like cell survival, proliferation and growth. Prior literature suggests reduced expression of AKT in SZ. mTOR is a kinase that forms 2 distinct complexes- mTORC1 and mTORC2. mTORC1 consists of mTOR, Raptor, GβL, PRAS40 and Deptor proteins. It plays an important role in actin dynamics and acts downstream of AKT. mTORC2 consists of mTOR, Rictor, GβL, Protor, mSin1 and Deptor proteins. It facilitates ribosome biogenesis and protein translation and acts upstream of AKT. Abnormalities in the mTOR complexes can contribute to dysregulated protein synthesis, which has been implicated in SZ. Alterations in the AKT-mTOR cascade, including abnormal phosphorylation of AKT and expression of mTOR complex components, have been suggested as potential mechanisms underlying SZ pathophysiology. Therefore, we hypothesized that protein levels and/or phosphorylation status of key molecules in the AKT-mTOR pathway are altered in SZ.

**Methods:**

We used post mortem dorsolateral prefrontal cortex (DLPFC) from 22 matched pairs of SZ and comparison subjects for this study. Using western blot analysis, we measured protein levels of AKT, mTOR, GβL, Raptor, phosphorylated AKT (at S473 & T308) and phosphorylated mTOR (at S2448 & S2481).

**Results:**

We found decreased levels of AKT, phosphorylated AKT (at both S473 and T308) and GβL. We also found that the ratio of phosphorylated mTOR (at S2448) to total mTOR was decreased.

**Discussion:**

AKT requires phosphorylation at both S473 and T308 for complete activation. It can further regulate the formation of mTORC1 through Rheb. AKT is phosphorylated at S473 by active mTORC2. mTOR phosphorylation at S2448 is required for its activation in both complexes. Our findings that total AKT and its phosphorylated forms are decreased in conjunction with reduced expression of GβL and the ratio of phosphorylated mTOR (at S2448) to total mTOR suggest that the AKT-mTOR signaling pathway is downregulated in SZ DLPFC. Given the importance of this pathway in synaptic plasticity via its regulation of protein translation and cytoskeletal organization, these abnormalities may represent a mechanism underlying cognitive dysfunction in SZ. Future studies will investigate the expression levels of proteins in mTOR complexes and will determine the integrity of mTORC1 and mTORC2 complex formation in SZ.

